# Bayesian Particle Instance Segmentation for Electron Microscopy Image Quantification

**DOI:** 10.1021/acs.jcim.0c01455

**Published:** 2021-03-08

**Authors:** Batuhan Yildirim, Jacqueline M. Cole

**Affiliations:** † Cavendish Laboratory, Department of Physics, University of Cambridge, J.J. Thomson Avenue, Cambridge CB3 0HE, U.K.; ‡ ISIS Neutron and Muon Source, STFC Rutherford Appleton Laboratory, Didcot, Oxfordshire OX11 OQX, U.K.; § Research Complex at Harwell, Rutherford Appleton Laboratory, Didcot, Oxfordshire OX11 OQX, U.K.; ∥ Department of Chemical Engineering and Biotechnology, University of Cambridge, J.J. Thomson Avenue, Cambridge CB3 0AS, U.K.

## Abstract

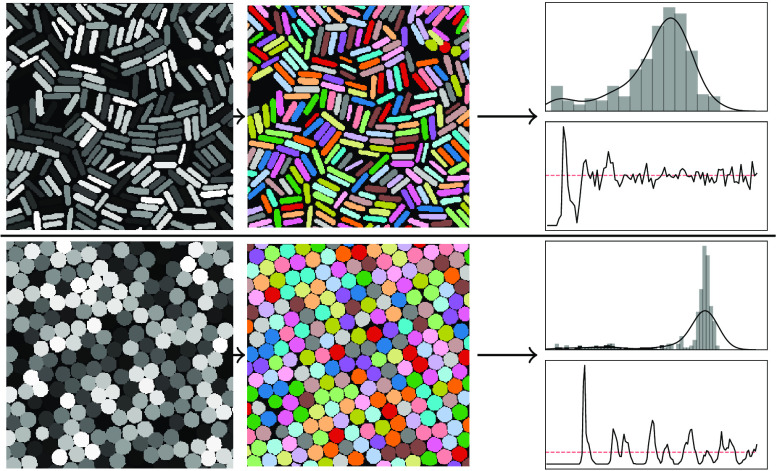

Automating the analysis portion of materials characterization by electron microscopy (EM) has the potential to accelerate the process of scientific discovery. To this end, we present a Bayesian deep-learning model for semantic segmentation and localization of particle instances in EM images. These segmentations can subsequently be used to compute quantitative measures such as particle-size distributions, radial- distribution functions, average sizes, and aspect ratios of the particles in an image. Moreover, by making use of the epistemic uncertainty of our model, we obtain uncertainty estimates of its outputs and use these to filter out false-positive predictions and hence produce more accurate quantitative measures. We incorporate our method into the ImageDataExtractor package, as ImageDataExtractor 2.0, which affords a full pipeline to automatically extract particle information for large-scale data-driven materials discovery. Finally, we present and make publicly available the Electron Microscopy Particle Segmentation (EMPS) data set. This is the first human-labeled particle instance segmentation data set, consisting of 465 EM images and their corresponding semantic instance segmentation maps.

## Introduction

The ability to automate analysis during characterization by electron microscopy (EM) is a desirable endeavor due to its capability to speed up particle analysis, as well as having the potential to collect data from EM images on a large scale. Measuring particle sizes from EM images has traditionally been performed using image-processing-based algorithms and this has proven an effective approach when doing so for a handful of images. However, the need to tune parameters to obtain accurate segmentations of particles in individual images makes this a highly manual approach on the small scale and a frail approach on the large scale. Learning-based segmentation methods that perform well in a variety of cases (given enough training data) have taken off in the past decade with the democratization of machine-learning methods in science. The robustness of these methods is well suited to automating data extraction and particle analysis from EM images, given that the need to tune parameters for edge cases can be eliminated by training on a sufficiently large and diverse set of labeled examples.

ImageDataExtractor^1^ was the first of its kind to introduce a pipeline to extract quantitative particle measures from EM images in a high-throughput manner. Its authors used a series of image-processing methods such as thresholding, contour detection, and ellipse fitting to detect and locate particles in electron micrographs, subsequently performing particle analysis on the identified particles. They were able to automate the entire process, from the extraction of EM images from scientific literature using ChemDataExtractor,^2^ to the measuring of scalebars in these images, achieving accurate particle and scalebar measurements. Similar to this work is that by Hiszpanski et al.,^3^ where image-processing techniques were employed to extract morphology and particle size information of nanoparticles from scanning electron microscopy (SEM) and transmission electron microscopy (TEM) images. This was part of a larger project in which nanomaterial synthesis procedures and information were extracted automatically from text and images available in the published scientific literature. Other examples of purely image-processing-based methods include work by Groom et al.,^4^ Meng et al.,^5^ and as well as Mirzaei and Rafsanjani.^6^ The former employs a thresholding-based segmentation approach, while the latter two use Hough transforms to segment particles following a series of preprocessing steps. Kim et al. measure particles in SEM images using a neural network to predict particle morphologies, followed by an application of the watershed segmentation algorithm to segment individual particles.^7^ Like ImageDataExtractor, they automatically detect the scalebar to obtain the pixel-to-nanometer conversion. They then measure particles by the length of line segments passing through the centers of each particle. Slightly different, but related work, by Tatum et al.^8^ is the Python library m2py, which performs semantic segmentation on scanning probe microscopy images, using a Gaussian mixture model (GMM) to identify a user-specified number of phases in the image. Once pixels belonging to the background and those belonging to particles/phases have been differentiated, m2py then performs instance segmentation using connected components labeling (CCL) or persistence watershed segmentation (PWS). They also show that instance segmentation can be performed directly using PWS, although by default this requires a single-channel image, meaning some form of dimensionality reduction is necessary for multichannel images. A commonality shared by these works (excluding m2py PWS and Kim et al.^7^) is that they perform a series of several steps to achieve segmentation. The drawback in this approach is that errors from the individual steps can accumulate to afford poorer results. Zhang et al.^9^ perform instance segmentation in a single step and measure particles from the resulting segmentations. They employed a deep-learning approach to segment particles using a Mask R-CNN^10^ instance-segmentation model, from which they were able to measure particles by performing edge fitting on the predicted segmentation maps. However, their model was trained on a small number of training examples (160), and their method is limited to spherical nanoparticles from TEM images, thereby lacking the diversity to be applied in a general sense for high-throughput use. Frei and Kruis^11^ also employ a Mask R-CNN model to segment particles for analysis, although their work differs in that they do so for agglomerated/partially sintered particles. Due to the difficulty in manually labeling data of such occluded particles, they train their model on synthetic data and show that their method performs better than standard Hough transform-based segmentation and ImageJ.^12^ Finally, Wu et al.^13^ also utilize Mask R-CNN to perform particle analysis in similar ways.

In this work, we present a Bayesian deep representation learning model for semantic particle instance segmentation and show that the method is effective in accurately segmenting individual particles. Like Zhang et al.,^9^ Frei and Kruis,^11^ and Wu et al.,^13^ we perform instance segmentation in a single step, thereby side stepping the problem of error accumulation. We train our model on the Electron Microscopy Particle Segmentation (EMPS) data set, a diverse collection of 465 electron micrographs and their corresponding human-labeled particle instance segmentation maps. While 465 samples may seem small as a training set for a deep-learning model, we show with model capacity experiments that we begin to reach performance plateaus with *n* < 465 training examples due to data augmentation. We show that our method performs significantly better than the previous image-processing approach, as well as the Mask R-CNN approach. We demonstrate the capability of our method to be used to perform particle analysis, by computing particle-size statistics, histograms, and radial- distribution functions from predicted segmentations. Finally, the method is integrated into ImageDataExtractor—a Python library for performing large-scale particle analysis on EM images.

## System Overview

### Particle Instance Segmentation

#### Basis of Model Formulation for Instance Segmentation

Modern representation learning-based instance-segmentation methods can be classified into two categories: proposal-based and proposal-free. The former consists of those methods where region proposals in the form of bounding boxes are produced from an input image, with segmentation subsequently being performed in these proposed regions.^15−18^ Since its inception, Mask R-CNN^10^ has been the prevailing deep-learning-based instance-segmentation method that falls into the proposal-based category. Based on a detect-and-segment approach, objects are first detected using a region proposal network (RPN), which proposes several regions of interest (RoI) to potentially be segmented. Then, for each proposed RoI, Mask R-CNN produces a binary segmentation mask, alongside bounding box offsets and object classifications. This approach proved highly effective and Mask R-CNN continues to be the dominant approach in instance segmentation. Although a powerful method, de Brabandere et al.^19^ highlighted the shortcomings of Mask R-CNN in cases where the objects being segmented overlap significantly. They argued, using a synthetic data set, that proposal-based methods fail to correctly segment overlapping instances in these cases, due to highly overlapping region proposal bounding boxes. Considering this evidence, along with the fact that highly overlapping nanoparticles are expected to be observed in EM images, this work takes a proposal-free approach for the task of particle instance segmentation.

In proposal-free methods,^19−24^ every pixel in the input image is mapped to a pixel embedding, such that pixels belonging to the same object/instance are encouraged to be close together in pixel-embedding space, while pixels belonging to different instances are pushed apart. This is usually achieved by designing a loss function that encourages grouping/clustering of pixels based on the *x*–*y* location and learned feature vectors of each pixel. Until recently, these methods suffered from their need to perform clustering using a separate algorithm during test time. This is disadvantageous as this can be slow on high-resolution images, and incorporating clustering into the loss function is likely to benefit the learned features of such a model for segmentation tasks. Neven et al.^20^ proposed a solution to this problem by outputting a seed map alongside pixel embeddings. Pixels with high seed scores in the seed map exist close to their instance centroid in embedding space, while those with lower scores are further away. Using this seed map, it is possible at test time to identify potential instance centroids and assign pixels to instances by determining to which centroid their embeddings lay closest.

**Figure 1:**
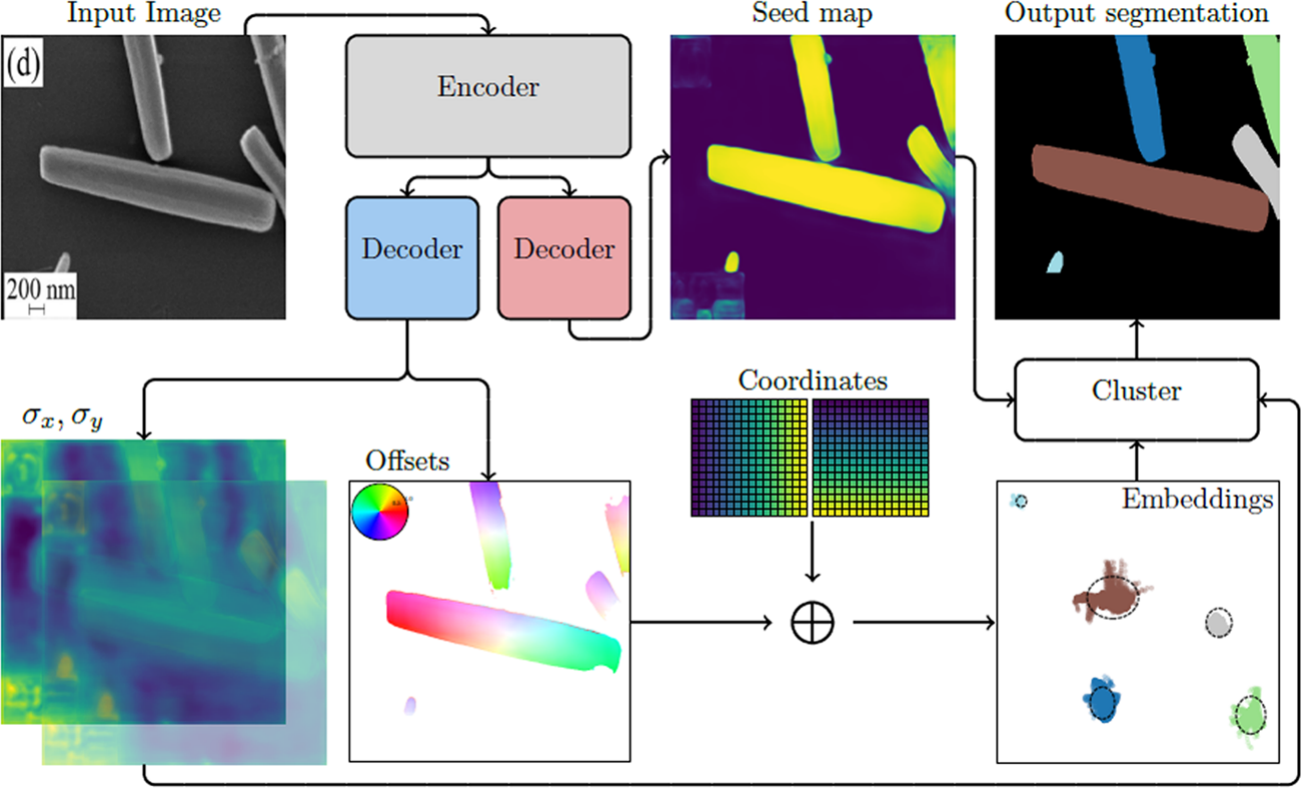
Pipeline for segmenting particle instances in EM images. An EM image is passed as input to the Bayesian particle instance segmentation (BPartIS) encoder to produce a latent representation of the input image. Standard deviations and offset vectors for each pixel are produced from this latent representation by the first decoder. Offset vectors are converted to spatially dependent pixel embeddings by adding the 2D coordinates of each pixel to each offset. The second decoder transforms the latent representation into a seed map, denoting which pixels are likely to be the centroid embeddings of each particle instance. The embeddings, standard deviations, and seed map are all used to cluster pixel embeddings to afford an output instance-segmentation map. The example used is an SEM image of ZnO microrods by Sarma and Sarma^14^ reprinted from ref (14), Copyright (2017), with permission from Elsevier.

This work adopts the semantic instance segmentation methodology of Neven et al.^20^ and recasts it in the framework of Bayesian deep learning. We set out to learn a function *f*(*X*; θ) that takes as input an EM image containing a set of particles and maps each pixel to an offset vector *o*_*i*_, from which we obtain their pixel embeddings *e*_*i*_. Each *o*_*i*_ is a two-dimensional (2D) vector that should point to the centroid of the particle instance, *S*_*k*_, to which the pixel belongs. Pixel embeddings are subsequently obtained by adding this offset vector to the 2D coordinate of each pixel in the input image *e*_*i*_ = *o*_*i*_ + *x*_*i*_, where *x*_*i*_ is the coordinate. By outputting offset vectors instead of embeddings directly, we avoid spatial equivariance by encoding the location of each pixel in the input image, thereby allowing the model to learn position-relative embeddings. This is crucial, since spatial-equivariant embeddings would mean that pixels belonging to two distinct particles that are identical in appearance but exist in different parts of the image would have the same *e*_*i*_ values. As a result, we would not be able to assign the pixels belonging to these particles to two separate instances. Hence, our function outputs offset vectors for each pixel, which we transform into spatially dependent pixel embeddings. This process is illustrated in Figure 1.

When learning our function, we desire that the mapping carried out by *f* will have the following properties. In pixel-embedding space, pixels belonging to the same particle instance should be close together. Additionally, clusters of pixels belonging to different particle instances should be well separated from each other. We model this function as a convolutional neural network (CNN) and learn its parameters θ using a supervised loss function, which encourages clustering of pixel embeddings that belong to the same class (particle instance). At test time, we subsequently use this function to compute the pixel embeddings of an input image and cluster them to assign each pixel to a particle instance (or background). We now describe the loss function used to learn θ and how this function achieves clustering of particle-instance pixel embeddings. The design choices of the model architecture that enable the use of this loss function are also outlined.

#### Learning to Segment Particle Instances

For each particle instance, *S*_*k*_, in the set of instances, *S*, we desire that each *e*_*i*_ ∈ *S*_*k*_ lay close to the instance centroid of *S*_*k*_. This way, we can assign pixels to particles in the input image based on to which instance centroid each pixel embedding is closest. During training, instance centroids, μ_*k*_, are computed by taking the mean of the embeddings that belong to an instance.
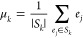
1

Hence, it would be possible to regress each embedding toward the centroid of the instance to which it belongs. In practice, however, minimizing a quadratic penalty to achieve this does not work so well when the objects being segmented vary in size. Larger particles are naturally comprised of a larger number of pixels in EM images, and it is likely that many of these pixels will exist far from the instance centroid in pixel-embedding space. These further pixel embeddings will dominate a loss function based on squared distance, leading to inferior performance.

Instead, this distance is converted into the probability of belonging to an instance, by placing an elliptical Gaussian kernel over the distance, ϕ_*k*_(*e*_*i*_)
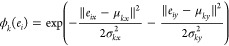
2

By letting our model output standard deviations, σ_*k*_, for each instance, larger σ_*k*_ values can be assigned to larger particle instances, meaning we overcome the previously mentioned issue of large particles dominating the loss function. Architecturally, we achieve this by outputting a σ_*i*_ for each pixel. We subsequently obtain σ_*k*_ by averaging each σ_*i*_ belonging to instance *S*_*k*_. During training, we encourage all σ_*i*_ values that belong to an instance to be similar to each other by including a smoothness term in our loss function. Finally, to determine the assignment of pixels to particle instances, we assign *e*_*i*_ to instance *S*_*k*_ if ϕ_*k*_(*e*_*i*_) > 0.5, and to the background or other instances otherwise.

Before defining the loss functions used to learn θ, there is an important architectural feature of the model that must be outlined. During training, we are able to obtain the centroids μ_*k*_ of each instance, since we train our model (supervised) using labeled data. We do so using the binary ground-truth segmentation maps as masks to obtain all pixel embeddings belonging to an instance, and average them. During test time, however, we do not have these centroids, and we must obtain them to cluster around each μ_*k*_. To achieve this, our model outputs a seed map (alongside offset vectors and sigmas), as described in the previous subsection and according to Neven et al.^20^ Pixels with a high seed score in the seed map lay close to the instance centroid, while pixels with lower seed scores are further away. This way, at test time, we identify the pixel embeddings with the highest seed scores and use these as proxies for our centroids, μ̂_*k*_. Then, for each μ̂_*k*_, we can find all pixels whose ϕ_*k*_(*e*_*i*_) > 0.5 and assign these to instance *S*_*k*_. We train our model to output the seed map such that background pixels are regressed to zero, while foreground pixels (those belonging to particles) are regressed to the output of the elliptical Gaussian function ϕ_*k*_(*e*_*i*_).

The loss function has three terms (and three weighting coefficients λ_*i*_) and is defined as follows
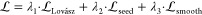
3

The first term is the Lovász-hinge loss,^25^ and its formulation is given by Berman and Blaschko.^26^ Minimizing this term directly optimizes the mean intersection-over-union (IoU) between the predicted and ground-truth segmentation masks of the particle instances. This encourages pixel embeddings to be close to their corresponding instance centroids in pixel-embedding space. We minimize the Lovász-hinge loss between the predicted ϕ_*k*_(*e*_*i*_) and ground-truth instance masks for each instance. The second term is the seed map loss
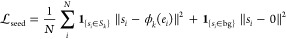
4where *s*_*i*_ are the seed-map values and the indicator functions denote binary masks for the foreground (particles) and background. This term regresses all background seed-map values to 0, and all foreground seed-map values to ϕ_*k*_(*e*_*i*_). Finally, the smoothness term encourages all σ_*i*_ ∈ σ_*k*_ values to be similar to each other
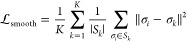
5

### Bayesian Inference

#### Basis of Bayesian Deep Learning Model Formulation

While deep neural networks are typically optimized to learn point estimates of their parameters, in cases where it is necessary to reason about uncertainty, it is favorable to take the Bayesian approach and instead learn distributions over the parameters of such models.^27−29^ In this paradigm, a prior distribution *p*(θ) is placed over the weights of the network. Given a data set with inputs **x** ∈ **X** and targets *y* ∈ **Y**, we seek to obtain the posterior distribution over the parameters using Bayes’ theorem
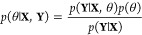
6where *p*(**Y**|**X**,θ) is the likelihood function and *p*(**Y**|**X**) is the marginal likelihood or normalizing distribution. If we were able to solve eq 6, we could obtain this posterior and perform inference by marginalizing over the parameter distribution
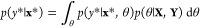
7

This amounts to evaluating a weighted average of the likelihood function for all possible θ, each weighted by its plausibility *p*(θ|**X**,**Y**).

In many cases, however, including the context of Bayesian neural networks (BNNs), performing inference this way is not possible, as the true posterior over the parameters cannot be evaluated analytically. Gal and Ghahramani^30^ proposed using variational inference to approximate this posterior, where an approximating variational distribution *q*(θ) is employed to estimate the true posterior. The form of *q*(θ) proposed by the authors is a Bernoulli distribution, which they showed can be conveniently modeled using dropout,^31^ a technique commonly used for regularization in neural networks. By minimizing the Kullback–Leibler (KL) divergence between *q*(θ) and *p*(θ|**X**,**Y**), the approximating distribution is encouraged to closely resemble the posterior. In practice, the KL divergence is implicitly minimized, since finding the optimal parameters of a dropout neural network is equivalent to finding the optimal variational parameters θ_*i*_ ∼ *q*(θ).^29^ By training a neural network with dropout, we can perform inference by approximating the marginal over the variational distribution using Monte Carlo (MC) sampling. This is achieved by first activating dropout at test time and performing *T* forward passes on the same input to obtain *T* MC samples. The average of these samples yields the MC estimate
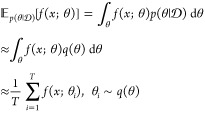
8where *f*(*x*;θ), the Bayesian neural network, is the likelihood function. The result of this is our MC estimate *p*_MC_(*y*|*x*). Thus, by implicitly minimizing the KL divergence between the posterior and the approximating distribution during training, it is possible to perform inference using a MC estimator to obtain predictions at test time.

#### Method Application of Particle Segmentation by Bayesian Inference

Once our model is trained, we predict particle instance segmentation maps from unseen EM images as follows. First, to perform inference, we output *T* MC samples by passing the same input through our model *T* times with dropout turned on, each time with different parameters due to the randomness induced by dropout. An individual MC sample (the output of a single MC estimator) consists of offset vectors (which we convert into pixel embeddings *e*_*i*_) and sigmas for each pixel in the input image, as well as a seed map denoting which pixels are likely to belong to particles. We obtain the final MC estimates from these outputs by averaging each component from the individual MC samples, yielding the MC estimates of embedding, sigma, and seed maps. Finally, we use these to cluster pixels and obtain the final instance-segmentation maps.

To cluster pixels, we obtain the maximum value in the MC seed map to find the pixel that is most likely to be the centroid embedding μ_*k*_ of the first particle instance. Once we know the location of this maximal value, we obtain the σ_*k*_ value of its instance from the MC sigma map. Armed with μ_*k*_ and σ_*k*_, we compute ϕ_*k*_(*e*_*i*_) for each pixel embedding, as in eq 2, and take all cases where ϕ_*k*_(*e*_*i*_) > 0.5 to be belonging to the first particle instance. We then obtain a binary mask of all pixels belonging to the first instance and use this to set all seed values in the seed map of this instance to 0. We can then find the next maximal seed-map value belonging to the next particle instance and repeat this until all particle instances have been identified.

### Quantifying Uncertainty

#### Statistical Basis of Uncertainty Quantification

The Bayesian approach has the advantage that it allows us to measure the uncertainties of the estimates of a model. The ability to measure the uncertainty of machine-learning-based operational pipelines can be crucial in informing the decisions that these systems make, as well as improving the quality of predictions. In this work, we make use of the uncertainties during the particle instance segmentation process, given that our end goal is to compute quantitative measures. Uncertain segmentations in our output segmentation maps are treated accordingly, such that they do not affect the quality of the quantitative measures that we compute. Specifically, any artifacts of the background, scalebars, or subpart labels that are incorrectly segmented as particles generally have high uncertainty and can easily be discarded and ignored in further computations.

Uncertainty can be distinguished into two distinct types: aleatoric and epistemic.^32^ Aleatoric uncertainty refers to the intrinsic noise in observations/data. In the case of human-labeled segmentation maps, a manifestation of this uncertainty would be due to errors in the edges of objects, where a few pixels of the background may have been included in the segmentation mask of an object due to human error. This type of uncertainty could theoretically be reduced if data labeling/collection could be done perfectly. However, this is generally not possible, and reduction in aleatoric uncertainty cannot be achieved by collecting more data. Epistemic uncertainty can be viewed as a model’s lack of knowledge, where its parameters are not well constrained to deal with out-of-distribution inputs. In these regions of input space, the posterior over the parameters is broad, resulting in high epistemic uncertainty. When performing Bayesian inference, this property of epistemic uncertainty allows one to discern whether the inputs provided to a model are out-of-distribution (with respect to the data the model was trained on), since such inputs will produce high epistemic uncertainties. This property can be used for outlier detection^33^ or may serve as an indication that the model would benefit from being trained with more data, since unlike aleatoric uncertainty, epistemic uncertainty can be reduced given more data.

Several methods have been proposed in the literature to compute uncertainty with Bayesian neural networks, which perform inference using MC sampling by dropout. One of these methods simply uses the entropy of the predictive distribution *H*[*p*(*y*|**x**)].^34^ Another simple method is to use the variance of the MC samples as a form of uncertainty.^30,35^ In this case, the idea is that the MC estimate of a model with low uncertainty will have low variance, and the model will confidently predict the same output more often than not, given an input and some paramaters sampled from an approximating distribution. Conversely, if the model is not paramaterized well to deal with an input, the individual MC samples will vary drastically and the variance will be high. While both straightforward to compute, the entropy of the predictive distribution does not distinguish model uncertainty from data uncertainty, while the variance is an approximation of model uncertainty. A more sophisticated method, and the one which we used in this work, is uncertainty based on the conditional mutual information (MI) between the output and the parameters.^36,37^ MI is a measure of the reduction in uncertainty in a random variable, after gaining knowledge of another. In the case of MI(*y*; θ|**x**), it measures the reduction in uncertainty of the parameters θ, given that we observe *y*; a high MI in this case indicates that knowing the true value of *y* would result in a large reduction in uncertainty, meaning that there is high uncertainty in θ at the onset. We express MI as the difference between: (1) the predictive entropy (total uncertainty) of the MC estimate, which acts as a proxy for the uncertainty in the marginal estimate of the model; and (2) the expected entropy (data uncertainty) that describes the average uncertainty of the individual MC samples, which make up the MC estimate.
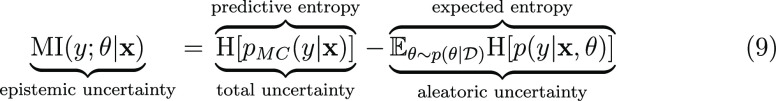
9

This way, we decompose the uncertainty into its epistemic and aleatoric components.^38,39^ In the case where the predictive entropy is similar to the expected entropy, the MI (and hence the epistemic uncertainty) is low, and we know that most of the uncertainty is aleatoric (due to data). In contrast, if the predictive entropy is high while the expected entropy is low, we know that epistemic uncertainty dominates the total uncertainty.

#### Method Application of Uncertainty Quantification

We are interested in modeling when an input lies in a region of data space that the parameters are not conditioned to deal with. Since data uncertainty is inevitable, we would like to explicitly model this and remove its contribution to the total uncertainty to obtain the model uncertainty. Smith and Gal^37^ showed that MI of *y* and θ is a measure of a model’s epistemic uncertainty, since being uncertain about an input *x* implies that if the model knew the true label *y* at that point, then information would be gained. In contrast, if our model parameters are well-conditioned to deal with *x*, we would gain little information in learning the true value of *y*. We therefore model the epistemic uncertainty in our outputs using MI, expressed in eq 9. By activating dropout at test time, we use the dropout approximation to obtain an MC estimate of the predictive distribution given an input **x**
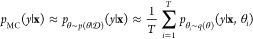
10where *p*_θ_*i*∼*q*_(θ)_(*y*|**x**, θ_*i*_) is a single MC sample—the output of a single MC estimator (our model with some weights randomly turned off by dropout). The entropy of *p*_MC_(*y*|**x**) gives us the predictive entropy (total uncertainty). We compute the expected entropy (aleatoric uncertainty) by taking the mean of the entropies of the individual MC samples. Putting these two together, we approximate the MI and hence the epistemic uncertainty by
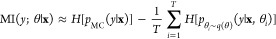
11

Recall that for each input image, our method outputs a seed map (alongside offset vectors and sigmas), which is an estimate of the probability ϕ_*k*_(*e*_*i*_) of each pixel belonging to a particle in the image. Thus, we can output *T* MC seed map samples and directly compute the predictive and expected entropies from these probabilities, followed by the conditional MI. This serves as the measure of epistemic uncertainty in our model outputs.

### Uncertainty Filtering

We use the uncertainties yielded by Bayesian inference to significantly improve the predictions of our model by identifying potential false positives and removing them. We simply compute the MI as mentioned in the previous subsection and use this as a measure of the epistemic uncertainty of our model given some input. From our initial particle-instance predictions, we can use the segmentation mask of each instance individually to obtain all uncertainty values that belong to an instance. By averaging the uncertainties for a predicted instance, we obtain the total epistemic uncertainty of that prediction. If this uncertainty is greater than some threshold *t*_u_, we label that prediction as a false positive and remove it from the final particle instance segmentation map. We use *t*_u_ = 0.0125 in this work for a model trained on 366 images from the EMPS training set. This value was found empirically—the details and results of this experiment can be found in the Results and Discussion section. The value of *t*_u_ will likely have to be tuned for models that are trained on greater or fewer data than this.

### Reducing Overfitting

Deep neural networks typically require several thousands of training examples to be able to approximate a function well. Nevertheless, several methods can be employed to circumvent this limitation when fewer data are available for the task at hand. Since the EMPS data set consists of a relatively small number of training samples for the context of deep learning, it is necessary to explore methods to overcome this limitation. These include simpler methods such as regularization and data augmentation,^40^ as well as more sophisticated methods such as transfer learning when sufficiently similar labeled data are available, and unsupervised pretraining when a large number of unlabeled data are available. The latter includes methods such as autoencoders trained to reconstruct their inputs,^41^ contrastive predictive coding,^42,43^ and information maximization.^44^

We found that data augmentation alone was enough to prevent overfitting, and more sophisticated methods were not necessary. When exploring the latter methods, we attempted unsupervised pretraining by image reconstruction (as well as data augmentation), due to its simplicity and to the availability of the SEM data set curated by Aversa et al.^45^ The SEM data set is an ideal candidate for two reasons: it consists of a diverse set of around 22 000 SEM images (without segmentation labels), around the order of magnitude needed to effectively train deep neural networks for vision tasks; the objects and textures found in these data are highly likely to share common characteristics with what is found in the EMPS data set. We trained a convolutional autoencoder to reconstruct the images from the SEM data set and used the encoder and decoder from this model to initialize our segmentation model (also an encoder–decoder architecture). Ultimately, we found that a model trained with a combination of unsupervised pretraining and data augmentation performed ever so slightly worse than one trained with data augmentation alone.

Thus, we opted for data augmentation without unsupervised pretraining. Augmentations used were: horizontal flips; vertical flips; random rotations; random color jitters (brightness ±0.3, contrast ±0.3, saturation ±0.3, hue ±0.3); random crops of half the original image resolution which were scaled up to the original resolution using bicubic interpolation for images, and nearest-neighbor interpolation for ground-truth segmentation maps.

### Implementation Details

#### Architecture

The underlying architecture of our model is an ERFNet^46^ with two decoders (one for offsets and sigmas, and one for seed maps) as per Neven et al.^20^ Offset vectors output by the first decoder are bounded between [−1, 1] using a tanh activation function, and the coordinate map added to this to obtain pixel embeddings ranges from [0, 1] in both *x* and *y* dimensions. Instead of outputting σ values directly, we output  and use an exponential function to obtain . A sigmoid activation function is applied to the output of the second decoder to obtain seed maps, since it is trained to produce probabilities ϕ_*k*_(*e*_*i*_) for foreground (particle) pixels and 0 for background pixels. We slightly modified the originally devised ERFNet^46^ in the following ways. We replaced all 2D transposed convolutions (deconvolutions) in the decoders with bilinear interpolation upsampling layers + standard 2D convolutions, since we initially observed checkerboard artifacts in our predictions as a result of the original transposed convolutions.^47^ Additionally, we replaced all rectified linear unit (ReLU) activations^48^ with exponential linear units (ELUs),^49^ as we found that this slightly improved performance.

#### Training

We trained our model for 300 epochs using the Adam^50^ variant of stochastic gradient descent with a learning rate of 0.0003. Weighting factors used in the loss function (as shown in eq 3) are λ_1_ = 1, λ_2_ = 1, and λ_3_ = 10. The training was performed on an NVIDIA Tesla V100 graphics processing unit (GPU) in Google Colaboratory, where a single epoch (training and validation) took roughly 70 s.

### Electron Microscopy Particle Segmentation (EMPS) Data Set

**Figure 2:**
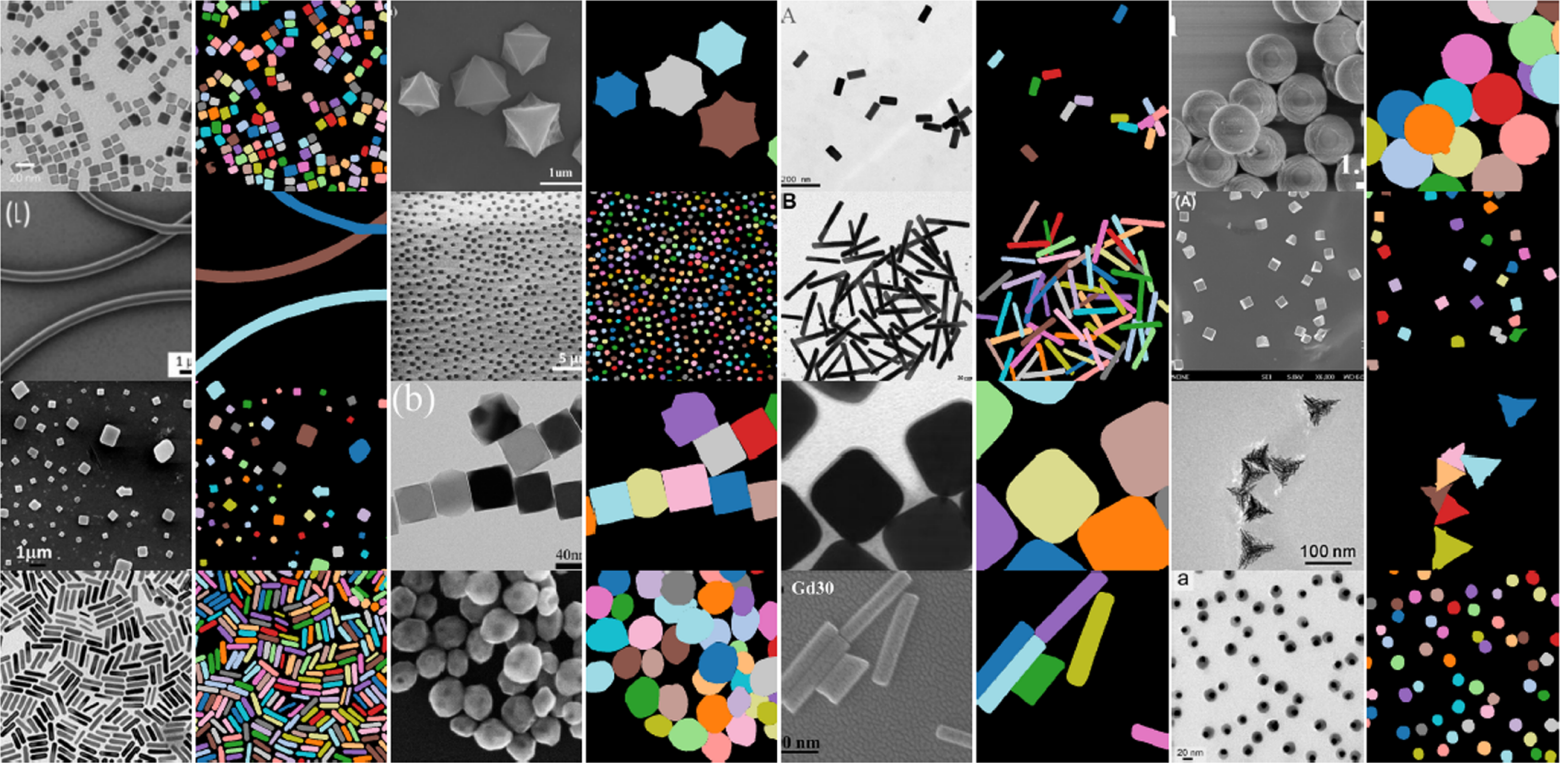
Sample images and corresponding instance-segmentation maps from the EMPS data set. Particle instances are denoted by the colored regions in the segmentation maps. Images going downwards then right are: Falcaro et al. reprinted from ref (51), Copyright (2016); Jiang et al. reprinted from ref (52), Copyright (2017); Navas and Soni reprinted from ref (53), Copyright (2016); Meng et al. reprinted from ref (54), Copyright (2017); Li et al. reprinted from ref (55), Copyright (2018); Balling et al. reprinted from ref (56), Copyright (2018); Yang et al. reprinted from ref (57), Copyright (2017); Distaso et al. reprinted from ref (58), Copyright (2017); He et al. reprinted from ref (59), Copyright (2019); Roy et al. reprinted from ref (60), Copyright (2017); Wu et al. reprinted from ref (61), Copyright (2020); Wu et al. reprinted from ref (62), Copyright (2017); Shang et al. reprinted from ref (63), Copyright (2020); Liu et al. reprinted from ref (64), Copyright (2017); Wang et al. reprinted from ref (65), Copyright (2017); and Wang et al. reprinted from ref (66), Copyright (2020). All with permission from Elsevier.

A bespoke Electron Microscopy Particle Segmentation (EMPS) data set was constructed to serve as the training data for this work. It consists of 465 electron micrographs and their corresponding human-labeled ground-truth semantic instance segmentation maps, as well as the coordinates of the polygons drawn around each particle to construct the segmentation maps. Figure 2 shows 16 sample images and their segmentation maps and portrays qualitatively the diversity of particle sizes, shapes, textures, densities, and (grayscale) colors that exist in the data set. Although not relevant for computing quantitative measures, we included several images where particles overlap each other with varying degrees of overlap, as this is common in the electron micrographs of nanoparticles. The third EM image in the second row of Figure 2 is an example of highly overlapping particles, while the particles in the fourth EM image of the third row show minor overlap.

All images in the EMPS data set were mined from published scientific literature using the data-mining application programming interface (API) of Elsevier. We first used the Article Retrieval API to obtain the digital object identifiers (DOIs) of articles published between the years 2015 and 2020, which had the possibility of containing EM images. This was achieved using the search query “SEM–TEM–scanning electron microscopy–transmission electron microscopy.” Next, using the Object Retrieval API, we iterated through the figures in these articles and obtained images at high resolution from any figure which contained one or more of the acronyms or phrases from our search query. This resulted in 34 091 images of figures, from which 788 were manually determined as suitable and set aside for postprocessing. It was often the case that EM images were part of a panel of several images in these figures. Thus, the EM images were cropped from these 788 figures, resulting in 962 potential images to be labeled (many figures contained several relevant EM images). We annotated 465 of these images using the VGG Image Annotator (VIA).^67,68^ This consisted of drawing polygons around each individual particle in each image. Once the annotation process was completed, we finally assigned pixels to particle instances in each image by finding all pixels that were encapsulated by the polygon of each particle.

**Figure 3:**
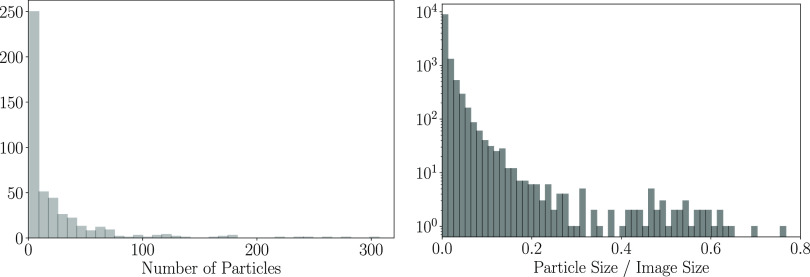
Image and particle-instance statistics from the EMPS data set. Left: number of particles per image. Right: particle-instance size as a percentage of image size (log *y*-scale).

Figure 3 presents some statistics of the images and particles in the EMPS data set. It is evident that most images contain fewer particles, and only a few images contain many particles (Figure 3, left). Similarly with particle sizes, most particles in the data set are small, with the number of large particles dropping significantly as particle size increases (Figure 3, right).

## Results and Discussion

### Technical Validation

#### Particle Instance Segmentation

We evaluate the performance of our Bayesian particle instance segmentation model (hereafter known as BPartIS) using a number of metrics and compare it to several similar algorithms as benchmarks. These include ImageDataExtractor,^1^ a Gaussian mixture model + connected component labeling^8^ and Mask R-CNN^10^ (with a ResNet-101 backbone^69^). To quantify the improvement in performance afforded by our Bayesian formulation, we also report the performance of the discriminative version of our method, as well as the Bayesian version for direct comparison. The EMPS data set was split into training and test sets consisting of 366 and 99 samples, respectively, where the former was used for training and validation, and the latter was used to evaluate our model, as well as the benchmark algorithms. Since there are cases where several EM images from a single publication exist in the EMPS data, the data were split such that images from the same publication were never spread across both the training and test set to avoid data leakage, i.e., multiple images from the same publication only appear in the training or the test set, but not both. We used average precision (AP) as defined by Hariharan et al.^70^ for our main evaluation metric. Given an input image, our model outputs a set of predictions for individual particle instances. We compare each of the individual predictions to all ground-truth particle instances. If the predicted particle instance segmentation mask and a ground-truth instance mask have an intersection-over-union (IoU) greater than some threshold *t*, we count this as a true positive. If a prediction has an IoU less than *t* with all ground-truth instance masks (there is no match), we count this as a false positive. Duplicates are defined as several predictions having an IoU greater than *t* for a single ground-truth instance. In this case, we designate the prediction with the highest IoU as a true positive, and the rest as false positives. Finally, a false negative is defined as any ground-truth instance which has not been matched with any predicted instance. We created a precision–recall curve by computing precision and recall at varying thresholds *t* and took the area under this curve as the average precision (AP). We also report AP_50_ and AP_75_, which are simply the precision values at *t* = 0.5 and 0.75. AP_75_ indicates how well the model performs under stricter true positive conditions and rewards methods with better localization. In addition to AP, we also report the mean IoU for all predicted particles
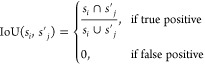
12where *s*′_*j*_ is the segmentation mask of the *j*th predicted particle instance and *s*_*i*_ is the *i*th ground-truth segmentation mask.

**Figure 4:**
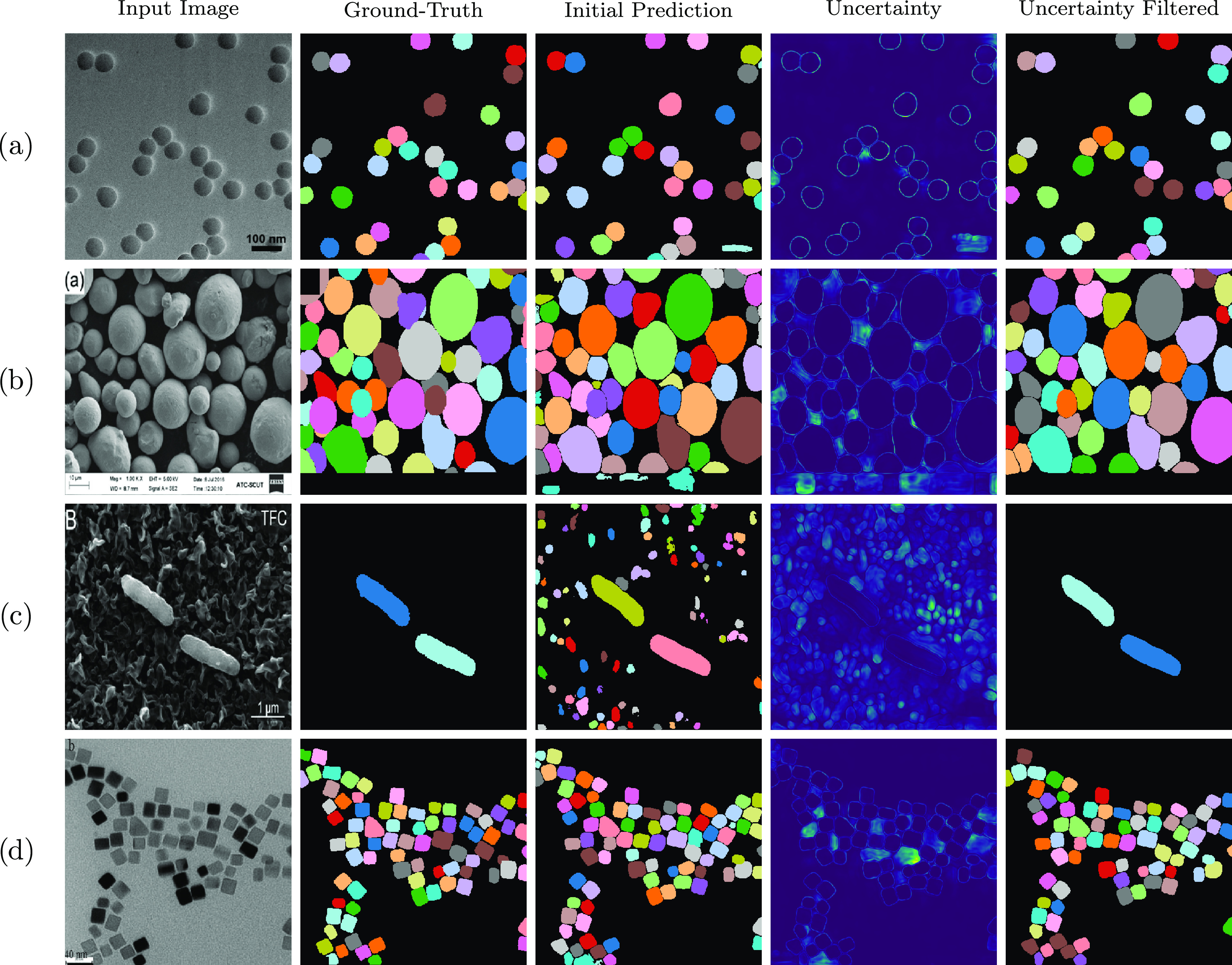
Qualitative results of performing Bayesian inference and uncertainty filtering with BPartIS on four examples from the EMPS test set. Predicted instance-segmentation maps and their corresponding uncertainty maps are shown, as well as the uncertainty-filtered final output. Notice how regions such as scalebars, text, and background textures are initially identified as particles with high uncertainty. These are subsequently removed to produce the uncertainty-filtered output, by removing all predicted instances with an uncertainty above some threshold *t*_u_. (a) TEM of functionalized silica nanoparticles by Sun et al.^71^ reprinted from ref (71), Copyright (2019); (b) SEM of grade 300 maraging steel powders by Tan et al.^72^ reprinted from ref (72), Copyright (2017); (c) SEM of bacterial cells by Faria et al.^73^ reprinted from ref (73), Copyright (2017); and (d) TEM of Pd cubic nanoparticles by Shah et al.^74^ reprinted from ref (74), Copyright (2017). All with permission from Elsevier.

**Figure 5:**
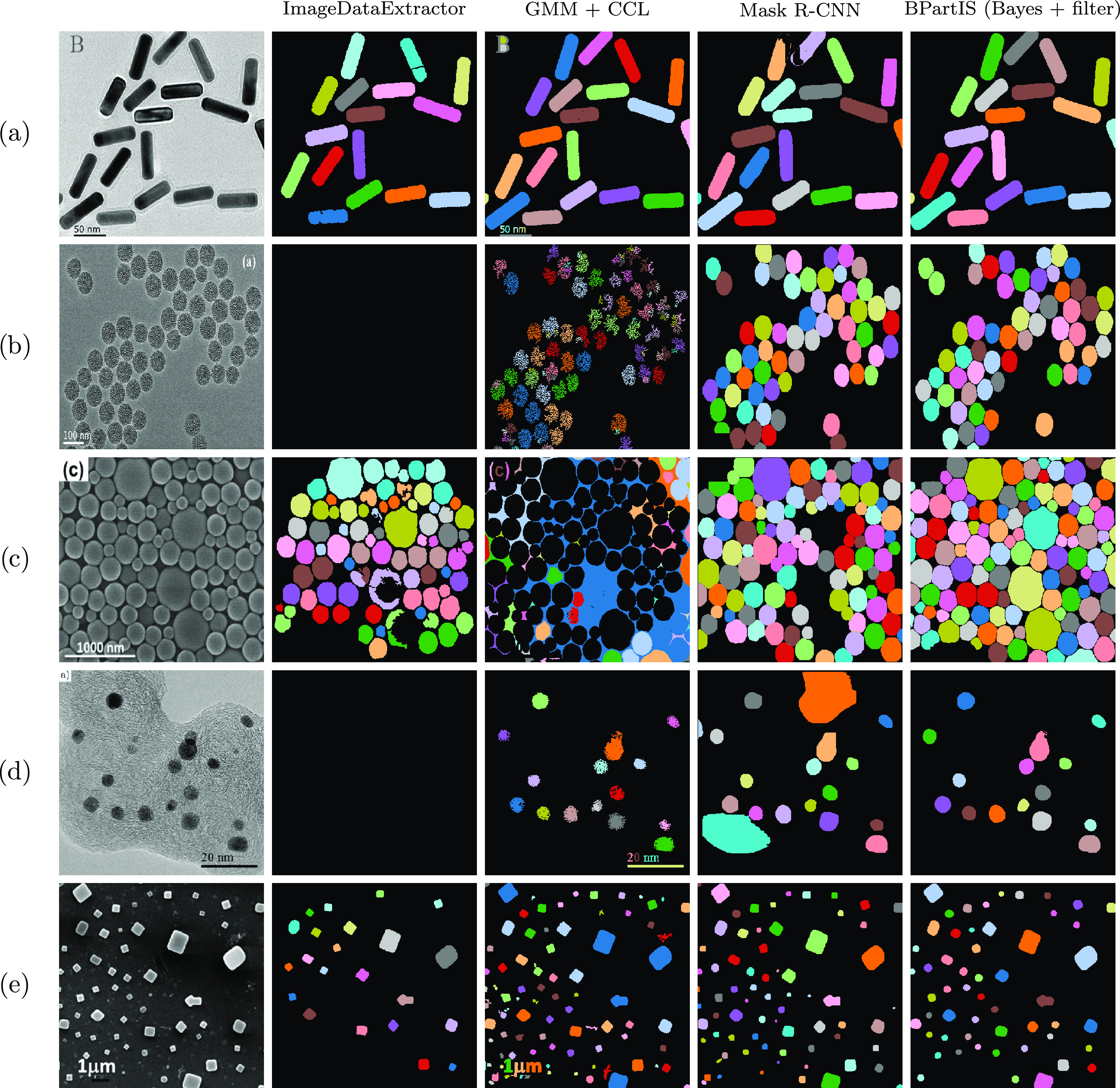
Qualitative comparison of BPartIS (Bayesian with uncertainty filtering) with other methods: ImageDataExtractor,^1^ m2py,^8^ and Mask R-CNN.^10^ All five images are from the EMPS test set. (a) TEM of Au nanorods by He et al.^59^ reprinted from ref (59), Copyright (2019); (b) TEM of dendritic-like mesoporous silica by Chen et al.^75^ reprinted from ref (75), Copyright (2020); (c) SEM of polydisperse polystyrene spheres by Zheng et al.^76^ reprinted from ref (76), Copyright (2020); (d) TEM of Pt_3_Co nanoparticles by Rasouli et al.^77^ reprinted from ref (77), Copyright (2017); and (e) SEM of Pd nanocrystals by Navas et al.^53^ reprinted from ref (53), Copyright (2016). All with permission from Elsevier.

The results in Table 1 show that BPartIS (Bayesian + filter) outperforms all of the benchmark methods on these metrics and significantly outperforms the image-processing and non-deep-learning-based particle segmentation methods. While all recent EM image quantification methods that employ learning-based models for segmentation use Mask R-CNN, these results suggest that proposal-free methods such as ours have greater potential for the task of particle instance segmentation. This is due to the inductive biases of proposal-free methods (pixel-wise clustering in a latent space) being more suitable for the domain of electron microscopy images, where objects can appear densely packed and overlapping. The results show that BPartIS (Bayesian), which performs prediction by Bayesian inference (without uncertainty filtering), results in a slight improvement over BPartIS (discriminative). This can be attributed to the fact that Bayesian inference does two things which result in improved performance: (a) a Bayesian model marginalizes over a posterior parameter distribution in which larger probability is assigned to more probable parameters given the observed data; (b) Bayesian models are ensemble methods; statistical ensembles are well-established as methods for improving prediction accuracy compared to single-estimator models. The significant increase in performance afforded by the Bayesian formulation combined with uncertainty filtering (BPartIS (Bayesian + filter)) is shown quantitatively in Table 1, and qualitatively in Figure 4, where the increase in precision is visually evident. Initially, regions such as scalebars and background textures are incorrectly segmented by BPartIS as particles (column 3 in Figure 4) but with high uncertainty (column 4 in Figure 4). Using the uncertainties of the individual predicted particle instances, we are able to filter out these false positives by their high uncertainties and achieve much higher AP, AP_50_, AP_75_, and mean IoU scores than the discriminative version (as well as all other benchmarks). The improvement in AP afforded by BPartIS (Bayesian + filter) relative to Mask R-CNN is evident in Figure 5, where the false positives predicted by Mask R-CNN do not appear in the segmentation maps predicted by BPartIS (Bayesian + filter). The results in Table 1 and Figures 4 and 5 suggest that the most significant gains in performance can be attributed to the ability of BPartIS (Bayesian + filter) to filter out false positives using uncertainties.

**Table 1:** Comparing the Performance of BPartIS (Our Method) to Similar Algorithms

method	AP	AP_50_	AP_75_	mean IoU
ImageDataExtractor	0.327	0.320	0.189	0.249
GMM + CCL	0.411	0.289	0.215	0.236
Mask R-CNN	0.506	0.668	0.638	0.621
BPartIS (discriminative)	0.560	0.786	0.738	0.712
BPartIS (Bayesian)	0.590	0.823	0.771	0.745
BPartIS (Bayesian + filter)	0.632	0.928	0.874	0.844

#### Analysis of the Uncertainty Threshold

**Figure 6:**
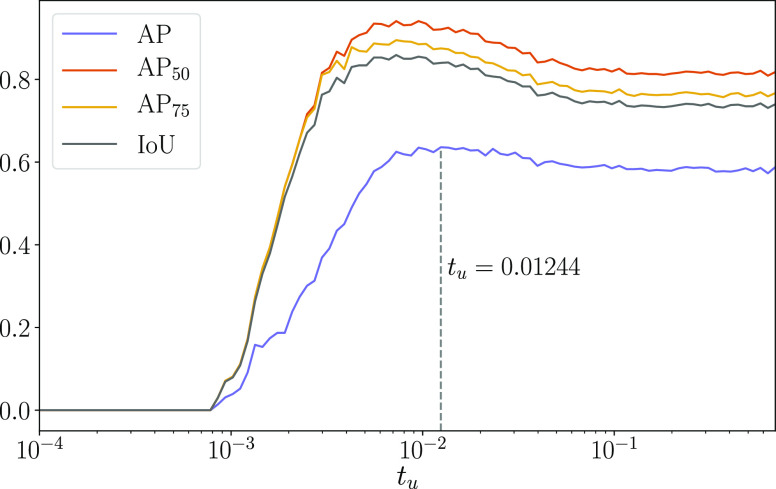
Metrics as a function of uncertainty threshold (*t*_u_).

We analyzed the effect of changing the uncertainty threshold on the performance of BPartIS (Bayesian + filter). To do so, we performed a gridsearch over 100 *t*_u_ values and examined the performance of the model on the AP, AP_50_, AP_75_, and mean IoU metrics. The results are shown in Figure 6. When the uncertainty threshold is low, all metrics become zero. Since BPartIS is unable to predict the segmentation mask of a particle with complete certainty, all predicted segmentations have some nonzero uncertainty, resulting in all particles being filtered out as false positives at low threshold values. This baseline uncertainty was found to be 7.8 × 10^–4^. Increasing *t*_u_ beyond this resulted in a steady increase in all metrics with AP peaking at *t*_u_ = 0.01244 and the other metrics peaking around *t*_u_ = 0.008 (*t*_u_ ranges between [0.0, ln 2]). Beyond these peaks, the performance drops slightly until around *t*_u_ = 0.09, leveling off as *t*_u_ increases beyond this for all metrics. At these postpeak values of *t*_u_, the drop in performance can be attributed to particles needing to have relatively high uncertainties to be filtered out, resulting in a higher false-positive rate. As a result of these findings, the default uncertainty threshold was selected to be *t*_u_ = 0.0125.

#### Model Capacity Experiments

**Figure 7:**
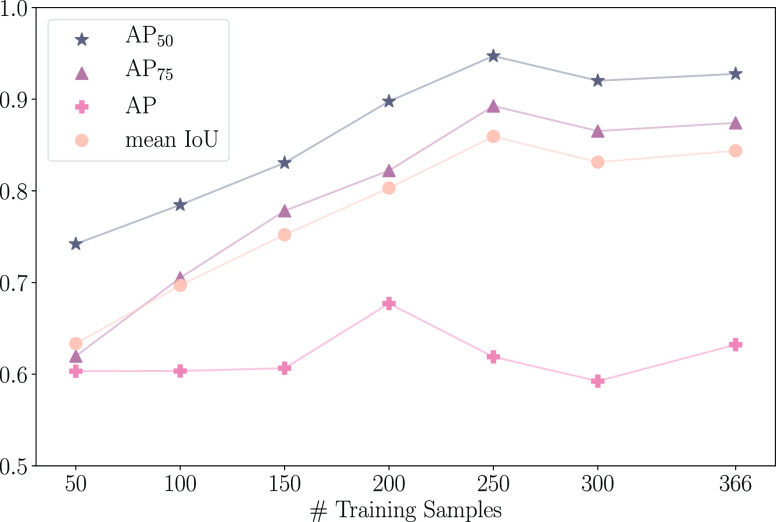
Metrics as a function of the number of training samples.

We investigated the capacity of BPartIS to improve as a function of the number of training examples. This is of interest due to the relatively small size of the EMPS data set. We trained several models with data augmentation using *N* ∈ {50, 100, 150, 200, 250, 300, 366} training examples in each case and evaluated each model on the same test set of 99 images. Figure 7 shows the performance of each model, where we initially observe a steady increase in performance by increasing the number of training samples. However, the increase in performance begins to level off after 200/250 training samples, and increasing the size of the training set beyond this does not improve performance significantly on the test set. These empirical results suggest that the use of data augmentation has allowed us to train an effective particle instance segmentation model with a relatively small number of training examples, and increasing the number of training samples further is unlikely to increase the performance enough to warrant further time and labor-intensive manual data labeling.

### Demonstration of Model in Automatic Particle Analysis

**Figure 8:**
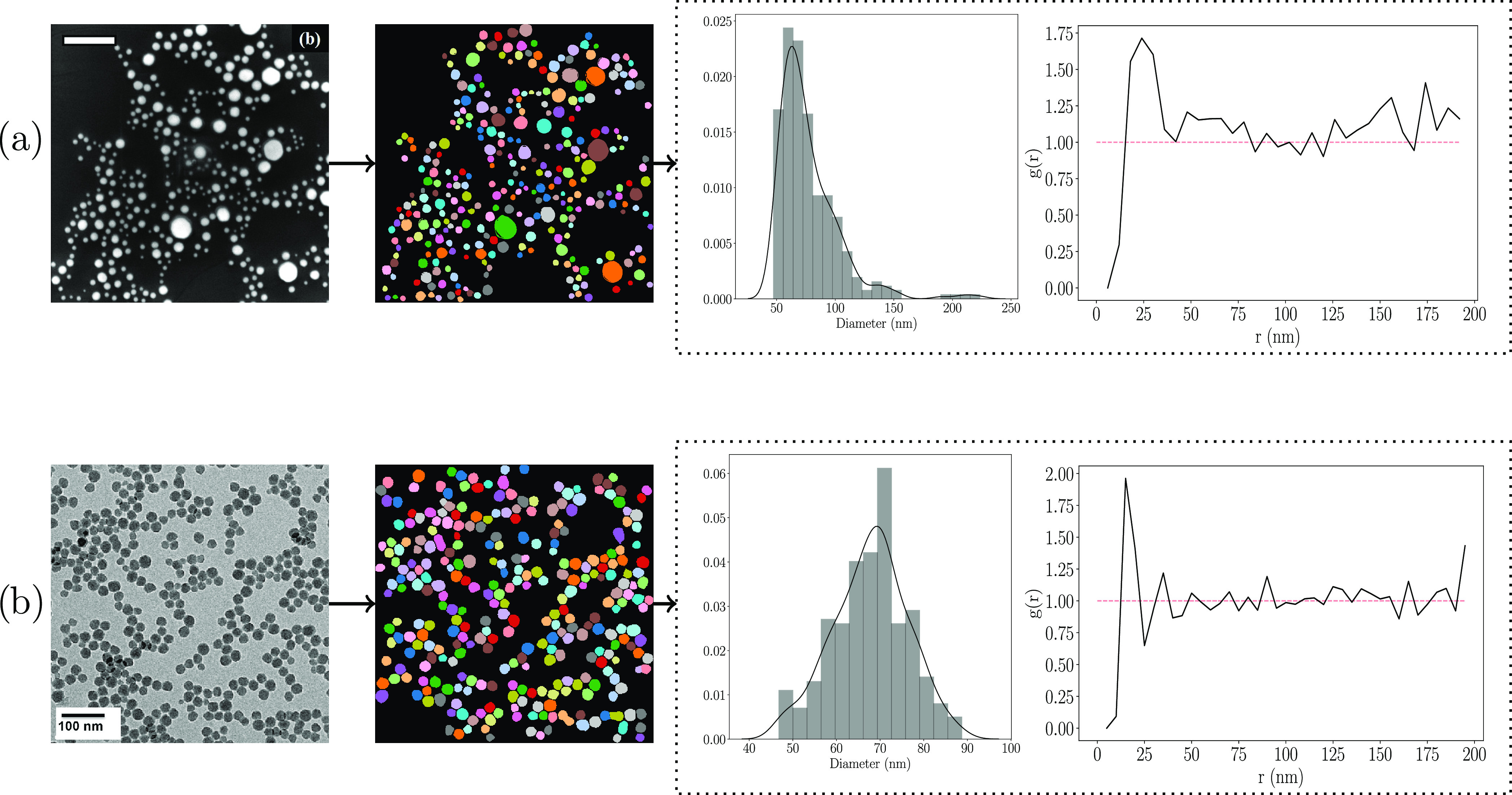
Example particle-size distributions and radial-distribution functions computed from BPartIS predictions of images not present in the EMPS data set. (a) SEM of Au@SiO_2_ core–shell nanoparticles by Gundanna et al.^79^ reprinted from ref (79), Copyright (2020); (b) TEM of ERM FD 304 colloidal SiO_2_ nanoparticles by Dazon et al.^80^ reprinted from ref (80), Copyright (2019). All with permission from Elsevier.

Our method can afford accurate and precise particle segmentation in an automated operational pipeline that measures and quantifies particles from EM images. Quantitative measurements that can be extracted from such a pipeline include average particle size, particle-size distributions, aspect ratios, and in some cases radial-distribution functions. As such, we present two case studies in which we employ BPartIS on EM images not present in the training or test sets and produce relevant quantitative measures of output. Although many works automate scalebar measurements to convert pixels into the relevant units (nm, μm), for the purpose of this demonstration, we measure them manually since in this work we are evaluating the particle segmentation capability of an automated particle measurement system. Besides, the strength of the BPartIS model is such that it has been incorporated into the ImageDataExtractor^1^ Python library, replacing the existing particle-detection and quantitative-analysis modules in v1.0, to form ImageDataExtractor v2.0. Therein, it uses the original automated scalebar-measurement modules. The particle analysis is illustrated in Figure 8. First, an input image is passed through the BPartIS model to obtain the initial instance-segmentation predictions and uncertainty map. The predictions are then postprocessed by uncertainty filtering to remove false-positive regions that may have incorrectly been classified as particles. Since the aim is to accurately measure particles, it is necessary to discard all predictions that exist on the border of the image, where it is likely that some proportion of these particles exist outside of the image. If these partially visible particles are not discarded, they will bias the aggregate measures or distribution of particle sizes if measured alongside the valid particles. Thus, we find all particles that intersect with the image borders and remove these from the set of particles to measure. Finally, we end up with a set of particles from which we can compute quantitative measures.

To measure each particle, we iterate through all of the predicted instance-segmentation masks and compute the area covered by each mask in pixels-squared. These measurements can subsequently be converted into relevant units by the conversion factor computed from scalebar measurements. From these, aggregate-size measures and distributions can be calculated. We show two examples in Figure 8 where we computed particle-size distributions and radial-distribution functions (using rdfpy^78^) from two images that are not part of the EMPS data set.

#### Quantitative Evaluation of Particle Analysis

Although the accuracy of the quantitative measures we compute can be extrapolated from the accuracy of the segmentation model, we performed an assessment of their accuracy by comparing particle analyses derived from BPartIS predictions with those derived from ground-truth segmentation maps from the EMPS test set. These include particle-size histograms, aspect-ratio histograms, and radial-distribution functions. For this analysis, we selected all images from the test set which contained greater than or equal to 30 particles, which resulted in 32 images. For particle-size and aspect-ratio histograms, we compared predicted to ground-truth histograms using the histogram cosine distance metric: , where *h*_1_ and *h*_2_ are the histograms being compared and *h*_1_(*i*) is the count of *i*th bin in *h*_1_. We compared predicted and ground-truth radial-distribution functions using the root-mean-square error (RMSE) metric: ∥*g*_pred_(*r*) – *g*_gt_(*r*)∥_2_, where *g*_pred_(*r*) and *g*_gt_(*r*) are the predicted and ground-truth radial-distribution functions, respectively. We computed these metrics for each of the 32 (prediction, ground-truth) pairs and averaged across all pairs. Average cosine distances for particle-size and aspect-ratio histograms were 0.02111 and 0.03261, respectively. The average RMSE for radial-distribution functions was found to be 0.63646. These values suggest that quantitative measures computed from BPartIS predictions can provide a faithful representation of the true underlying particle statistics present in an EM image.

## Conclusions

We have presented a Bayesian deep-learning methodology to segment particle instances in EM images for quantitative analysis. By comparing the performance of BPartIS with other similar methods, we showed that our method performs better than an image-processing method, a machine-learning-based method, and a proposal-based deep-learning method for particle instance segmentation. Our model was trained on a human-labeled particle instance segmentation data set consisting of 465 images and ground-truth segmentation maps, which we make publicly available at https://imagedataextractor.org/evaluation. We have demonstrated the ability of BPartIS to be used in quantitative particle analysis, by computing particle-size distributions and radial-distribution functions from EM images found in scientific literature. The code and implementation of BPartIS can be found at https://github.com/by256/bpartis, where we provide scripts to reproduce our results. The BPartIS model replaces the particle-detection and quantitative-analysis steps of ImageDataExtractor^1^ to afford ImageDataExtractor v2.0.

## Notes

ImageDataExtractor 2.0 and BPartIS are released under the MIT license and are available to download from https://imagedataextractor.org, where an interactive demo, user guide, code examples, and full API documentation are available. The Electron Microscopy Particle Segmentation (EMPS) data set is available at https://imagedataextractor.org/evaluation, which includes 465 images and ground-truth segmentation maps.
